# Review of the Pathology of Muscle in Amyotrophic Lateral Sclerosis

**DOI:** 10.3390/ijms27062802

**Published:** 2026-03-19

**Authors:** Matthew Katz, Thomas Robertson, Shyuan T. Ngo, Sai Yarlagadda, Robert D. Henderson, Pamela A. McCombe, Peter G. Noakes

**Affiliations:** 1 Department of Neurology, Royal Brisbane and Women’s Hospital, Herston, QLD 4006, Australia; matthew.katz@health.qld.gov.au (M.K.); robert.henderson@health.qld.gov.au (R.D.H.); 2 Anatomical Pathology, Pathology Queensland, Herston, QLD 4006, Australia; thomas.robertson@health.qld.gov.au; 3 School of Biomedical Sciences, The University of Queensland, St. Lucia, QLD 4072, Australia; s.ngo@uq.edu.au (S.T.N.); s.yarlagadda@uq.edu.au (S.Y.); 4 Australian Institute for Bioengineering and Nanotechnology, The University of Queensland, St. Lucia, QLD 4072, Australia; 5 Centre for Clinical Research, The University of Queensland, Herston, QLD 4006, Australia

**Keywords:** amyotrophic lateral sclerosis, motor neuron disease, muscle, pathology

## Abstract

In amyotrophic lateral sclerosis (ALS), a central event is the withdrawal of the motor nerve terminal from its target muscle. Whether this defect is driven by faults in the motor neuron or faults that originate within the muscle remains an area of investigation. In this review, we focus on the pathological abnormalities that are found in skeletal muscle, focusing, when possible, on human ALS, with support from ALS animal models. We begin with an overview of skeletal muscle, including a review of muscle fiber type, motor units and the neuromuscular synapse. Next, we provide a description of the clinical and biomarker changes that occur in the muscles of patients with ALS. We provide an extensive account of the histopathological changes that are evident in ALS muscle, such as fiber type grouping, muscle inflammation, protein misfolding, mitochondrial dysfunction, and alterations in neuromuscular junctions and muscle satellite cells. Our review then concludes with an update of metabolic and molecular–genetic changes that are found in ALS muscle. The evidence shows that muscle can be an additional target for therapy in ALS, in combination with therapies targeting neurons and glia within the central nervous system (CNS).

## 1. Introduction

Amyotrophic lateral sclerosis (ALS) is a neurodegenerative disease characterized pathologically by the loss of neurons of the motor cortex and alpha-motor neurons (α-MNs) of the spinal cord and brain stem [1,2]. The cause of ALS is still unclear. Possible pathogenic mechanisms include glutamate toxicity, abnormal RNA processing, accumulation of abnormal protein aggregates and abnormalities of autophagy. It is thought that the development of ALS is a multi-step process [3,4]. The risk of developing ALS is due to both environmental and genetic factors, with genetics estimated to contribute about 61% of the variance in the risk of developing ALS [5]. ALS is usually considered to be a disease of aging; however, more needs to be known about its pathogenesis.

The role of genetics in ALS is complex. There are some single genes that have variants that are strongly associated with ALS [6]. Of these, the most common is repeat expansions of chromosome 9 open reading frame 72 (*C9orf72*). While *C9orf72* is not a new gene, its pathogenic expansion is considered to be relatively recent in human evolution, having arisen from a common founder some 1500 years ago in the Scandinavian region [7]. There are also other genes of small effect that contribute to the risk of developing ALS, as shown in genome-wide studies [8]. Studies also indicate that genetic variation can influence the severity and age of onset of disease [9]. There appear to be interactions of genes for metabolism with the risk of ALS: for example, *GPX3*, which encodes for glutathione peroxidae 3 (GPX-3) is downregulated in ALS muscle and plays a role in regulating oxidative stress. GPX-3 is also known to interact with known ALS-linked genes, such as *SOD1* [10], and is identified as an ALS-risk gene [11]. This is a field that is rapidly expanding, but more needs to be known about the genes that increase the risk of ALS, and those that influence the severity of the disease, with a view to using this knowledge to advance therapy.

There has been recent interest in evolution and ALS. ALS occurs only in humans. It has been pointed out that ALS affects the newest brain functions that have evolved with the development of humans [12]. Recently, it has been suggested that the development of these brain regions was associated with the loss of the *CMAH* gene in humans [13]. The loss of this gene had a survival advantage in resistance to certain infections. This fascinating field of study is expanding and helps to provide insight into how genes may have been selected for benefit in early life but were not beneficial in aged people.

Although ALS occurs only in humans, animal models have been developed in many species (see review [14]). The most common models are transgenic mouse models, which have inserted human genes that carry known ALS mutations—the most common being the SOD1G93A mutation [15]. These models do not fully reproduce human disease, possibly because rodent brains differ from human brains, as they are developed in inbred strains, and because the insertion of multiple copies of a human ALS transgene represents a gain of toxic function. Another feature not generally appreciated is the morphological differences between rodent and human neuromuscular junctions (NMJs) that have bearing on NMJ function [16]. Rodent NMJs are bigger and have a “pretzel” shape morphology compared to human NMJs, which are significantly smaller and possess an “en grappe” morphology [17]. Collectively, these drawbacks and differences need to be considered when trying to apply the results of laboratory rodent models to ALS in humans.

ALS is characterized pathologically by loss of motor neurons, but the key clinical finding is weakness due to denervation of muscles through withdrawal of neuronal terminal branches from NMJs [18]. In addition, there could be additional roles for muscle in ALS, and some have argued for a more direct contribution to ALS pathogenesis [6,19,20,21,22]. In this review, we report the abnormalities of the muscle described in ALS. We first describe the features of muscle that are important to ALS and then review the evidence from muscle biopsies from people with ALS. Next, we review the possible causes of muscle abnormalities in ALS, with evidence from human studies and from laboratory models. The overall aim of the review is to consider the strength of the evidence for the accepted dogma concerning the role of muscle in ALS.

## 2. Overview of Skeletal Muscle Structure and Fiber Types

**Figure 1:**
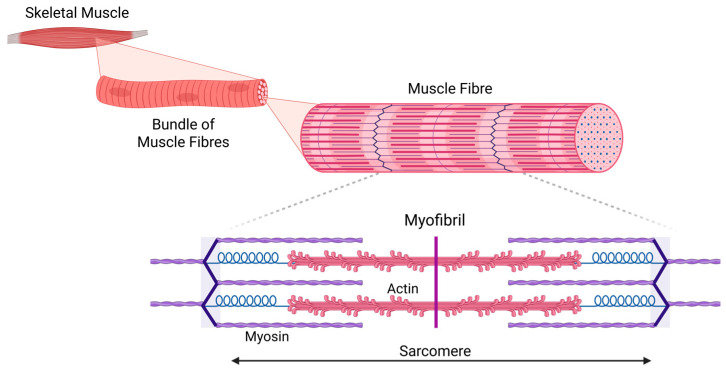
Hierarchical structure of skeletal muscle and sarcomere organization. Skeletal muscle comprises bundles of muscle fibers (myofibers), which are multinucleated cells formed through the fusion of myoblasts. Each muscle fiber contains longitudinally arranged myofibrils, composed of repeating sarcomeres that serve as the basic contractile units of muscle. In the magnified panel, thick filaments (myosin) are centrally located, while thin filaments (actin) extend from the Z-lines toward the center. Myosin heads interact with actin filaments to facilitate contraction via the sliding filament mechanism. This schematic illustrates the structural organization of muscle from the tissue level to its molecular components. Created in BioRender. Yarlagadda, S. (2026) https://BioRender.com/g1c41uu (accessed on 9 March 2026).

Skeletal muscle is made up of bundles of muscle fibers (myofibers) distributed randomly within fascicles [23]. Muscle fibers are syncytial cells, formed by the fusion of individual myoblasts [24,25,26], and within each myofiber, overlapping myosin and actin filaments create sarcomeres, the basic contractile unit of muscle [25] (Figure 1). Muscle fibers are innervated by α-MNs, resulting in a synapse, termed the neuromuscular junction (NMJ). Muscle contraction is facilitated by the release of calcium ions (Ca^2+^) from the sarcoplasmic reticulum (SR) following the generation of muscle action potential by α-MNs through the NMJ, a process known as excitation–contraction coupling [25]. Excitation–contraction coupling is a series of events that begins with the muscle action potential and ends with the activation of the intracellular dihydropyridine receptor to release intracellular Ca^2+^ to trigger actin–myosin-mediated muscle contraction [27]. These physiological mechanisms are well established and are fundamental to our understanding of how weakness can occur.

As first conceived by Sherrington, a motor unit is composed of the α-MN, its terminal axon branches and all the muscle fibers that it innervates [28,29]. The muscle fibers of a motor unit are intermingled with muscle fibers from other motor units [30,31]. Skeletal muscle contains different kinds of motor units that function in postural control and movement. In human muscle, there are three types of motor units: Type S (slow twitch), Type FF (fast fatigable) and Type FR (fast, fatigue resistant) [32,33]. Fast fatigable (FF) motor units rely on glycolysis for energy (i.e., ATP) and are innervated by large α-MNs that are less excitable than other α-MNs and display a burst frequency of action potentials [34]. Fast fatigable-resistant (FR) motor units rely on both glycolysis and oxidative pathways to generate ATP, while slow motor units rely only on oxidative pathways. Since the pathology of ALS includes loss of anterior horn cells, weakness occurs as individual motor units are lost.

FR motor units are characterized by smaller α-MNs that have a high tonal frequency of action potentials, while slow motor units are characterized by small α-MNs which have a slow tonal frequency of action potentials [35,36]. In voluntary muscle contraction, motor units are recruited in an orderly fashion, starting with Type S units, followed by FR and then FF fatigable units [37]. The basis of this orderly recruitment emanates from the orderly activation of α-MNs within a motor pool (all the α-MNs that innervate a muscle), beginning with small α-MNs through to larger α-MNs [37,38], commonly referred to as “*Henneman’s size principle*”.

While motor units are categorized as fast or slow, and according to resistance to fatigue, muscle fibers can be categorized as Type I (slow twitch) and Type II (fast twitch), with Type II fibers being split into two groups (IIA and IIB). These muscle fiber types were originally distinguished by ATPase staining [39] but are now defined by the presence of different isoforms of myosin heavy chain (MHC) [31,36,40]. The type of MHC in muscle determines the shortening velocity of the fiber [41], so the type of MHC in a fiber is expected to determine whether the fiber shows slow or fast twitch properties. Similarly, the proportion of different fibers in a muscle determines whether it is a postural muscle or is mostly used for movement. This is well known in the case of the muscles of the lower limb, where the *Gastrocnemius* muscle have more fast fibers than the *Soleus*, which is a postural muscle [42], and where the *Soleus* and *Gastrocnemius* show different patterns of activation [43].

The muscle fiber type is related to the properties of the α-MN by which it is innervated, as first shown by direct recording from motor axons [44]. Studies in the cat found that all the muscle fibers of a motor unit are of the same type [45], further indicating that muscle fiber type is determined by the type of nerve fiber that innervates muscle [46]. This has now been shown to be the case in most mammals [47], including humans [48]. Early experimental studies also confirmed that muscle fiber type is related to innervation [49,50]. This dependence of muscle fiber type means that loss of motor units of a given type will result in the loss of the type of muscle fibers that are dependent on that type of motor unit. This means that loss of a given muscle fiber type will result in changes in the proportions of different muscle fiber types in a muscle. This is important in ALS, where there is thought to be a different vulnerability of different types of motor units.

The mechanism by which α-MNs regulate muscle fiber types is thought to be via differences in frequency and duration of nerve stimulation of the NMJ by different types of anterior horn cells [36,41,51,52]; this is determined by the size of the soma of the anterior horn cell [53]. It appears that different rates of stimulation lead to variation in intracellular calcium levels; this in turn affects the activity of transcription factors such as the nuclear factor of activated T cells (NFAT) and second messages such as AMPK (AMP-activated protein kinase), SIRT1 (Sirtuin 1) and PGC1α (Peroxisome proliferator-activated receptor-gamma coactivator-1 alpha), leading to alteration in myogenic gene expression [31,41]. NFAT has been identified as a key effector pathway for muscle fiber type switching in response to different motor neuron firing patterns [31,54]. These important molecules deserve further study as possible targets for intervention in ALS.

The dependence of muscle fiber type on nerve fiber innervation is supported by the phenomenon of fiber type grouping, when previously denervated muscle fibers undergo a change in muscle fiber type according to the type of re-innervating motor neuron [55,56]. However, this is not always perfect and sometimes results in muscle fibers of intermediate type [56,57]. There is also the possibility of some influence of the muscle fiber on the neuron, with some muscle fibers resisting re-innervation [58]. Fiber type grouping has been considered a key finding in pathological studies of ALS muscle (see below).

**Figure 2:**
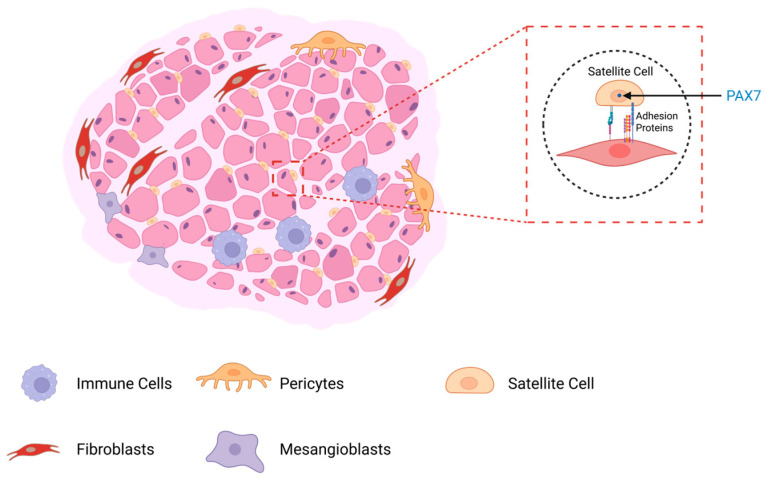
Cellular composition of skeletal muscle and localization of muscle satellite cells. In addition to myofibers, skeletal muscle contains several other cell types, including satellite cells (muscle stem cells), immune cells, fibroblasts, pericytes, and mesangioblasts. These cells play a crucial role in tissue maintenance and regeneration. Satellite cells, located beneath the basal lamina but outside the sarcolemma, are the primary stem cell population responsible for muscle repair following injury. They are identified by high expression of the transcription factor PAX7, which maintains their quiescence under normal physiological conditions. Created in BioRender. Yarlagadda, S. (2026) https://BioRender.com/hsi5qor (accessed on 9 March 2026).

Skeletal muscle also contains other types of cells, including muscle stem cells (termed satellite cells), immune cells, fibroblasts (connective tissue cells), and rare cells that can, under certain physiological conditions, aid in the repair of muscle cells (e.g., pericytes and mesangioblasts) [24,59]. Muscle satellite cells, which lie under the basal lamina but exterior to the sarcolemma, are the best characterized muscle stem cells and are important for muscle regeneration after injury [60,61] (Figure 2). Satellite cells are identified in muscle by their high expression of Pax7, a helix-loop transcription factor that helps to prevent them from entering myogenesis under normal physiological conditions [24]. The upregulation of myogenic transcription factors such as MyoD with a corresponding downregulation of Pax 7 allows satellite cells to progress along the myogenic pathway [62].

**Figure 3:**
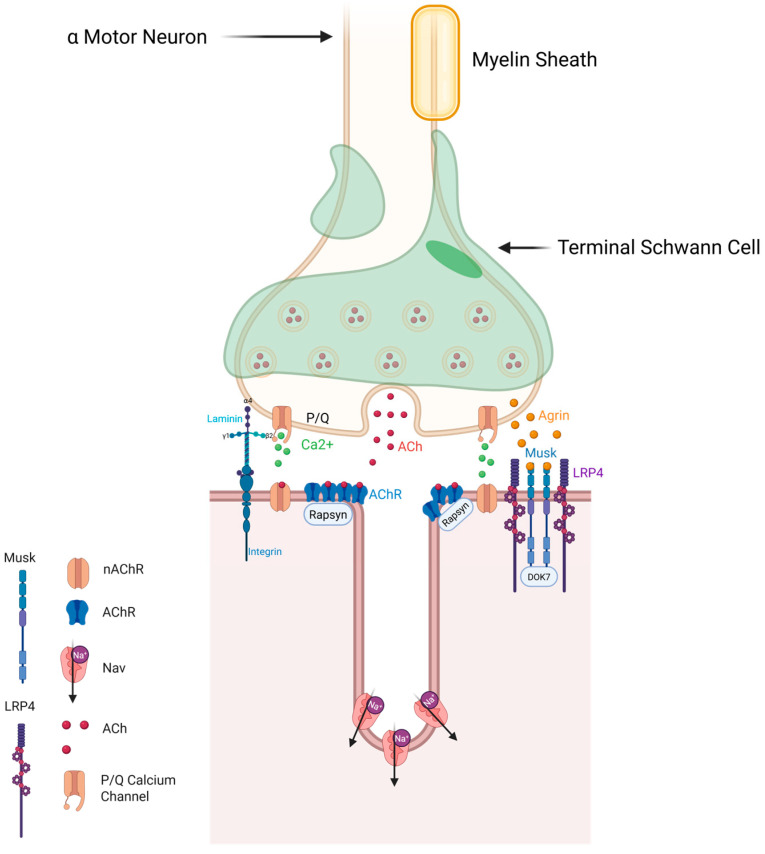
Structure and molecular features of the neuromuscular junction (NMJ). The NMJ is a specialized synapse where α-motor neurons connect with skeletal muscle fibers. It includes the presynaptic nerve terminal, the synaptic cleft, and the postsynaptic muscle membrane, which is enriched with acetylcholine receptors (AChRs). These receptors are clustered by rapsyn and stabilized by the LRP4–MuSK–DOK7 signaling complex. The synaptic cleft contains a specialized basal lamina with molecules such as synaptic laminins (Laminin) and agrin. Agrin binds to LRP4 and activates MuSK, promoting AChR clustering and maintaining the NMJ. Junctional folds in the postsynaptic membrane concentrate AChRs at the crests and voltage-gated Na^+^ channels (Nav) in the troughs, enhancing synaptic efficiency. Terminal Schwann cells, shown capping the nerve terminal, contribute to synaptic maintenance, repair, and plasticity. Created in BioRender. Yarlagadda, S. (2026) https://BioRender.com/t4q65dl (accessed on 9 March 2026).

The NMJ, where terminal axonal branches of α-MNs synapse with muscle, is a complex structure, formed by the distal axonal terminal (motor nerve terminal), the synaptic cleft and the underlying muscle membrane, which bears acetylcholine receptors (AChRs) in very high density [63] (Figure 3). This underlying membrane is also enriched by NMJ proteins, such as LRP4, MuSK, and DOK7, that are needed for NMJ stability [64,65]. The synaptic cleft (the gap between the motor nerve terminal and underlying muscle membrane) is filled with basal lamina, which is composed of selective NMJ induction, adhesion and signaling molecules, such as the synaptic laminins (α4-, α5-, and β2-laminins) [66,67,68,69,70,71,72] and the heparin sulphate proteoglycan agrin [73,74], both of which are required for the formation and maintenance of NMJs [75].

Agrin binds to the LRP4-MuSK-DOK7 complex to assist with stabilization and growth of high-density AChRs in the muscle membrane [76,77,78]. The postnatal infolding (junctional folds) of the muscle membrane under the motor nerve terminal allows for further concentration of AChRs at the tops of these NMJ folds, along with a concentration of voltage-gated Na^+^ channels along the valleys of these folds [16] (Figure 3). Human NMJs, whilst smaller than other mammalian NMJs such as that of the mouse, have some of the deepest junctional folds, making it a very efficient synapse despite its smaller size [16,17]. The remaining cellular element of the NMJ is the terminal non-myelinating Schwann (glial) cell (Figure 3) [79]. These cells are involved in modulating NMJ synaptic transmission, NMJ plasticity and NMJ repair following denervation [80,81,82,83]. There is increasing interest in NMJ remodeling dynamics in ALS [18,84,85], and interest in the NMJ as a therapeutic target.

Recent studies have shown that overexpression of either MuSK or DOK7 in skeletal muscle of ALS model mice can dramatically slow the rate of muscle denervation, and in the case of DOK7, extend the life span of SOD1^G93A^ ALS model mice [86,87]. Current studies are now developing humanized muscle-specific AAV-DOK7 for human treatments (e.g., see [88]). In the case of MuSK, recent research has led to the development of a human agonist anti-MuSK (ARGX-119) designed to stimulate MuSK, which in turn has been shown to stabilize the NMJ, and thus delay muscle denervation of muscle in ALS model mice (e.g., SOD1G93A and C90rf72 mice [89,90,91]). These findings for MuSK have led to further clinical development of ARGX-119 as an NMJ stabilizing treatment for ALS. Phase 1 clinical trials have met with success, and ARGX-119 is now undergoing Phase 2a reALiSe study with results expected in 2026-2027 (Argenex, https://clinicaltrials.gov/study/NCT06441682, accessed on 17 September 2025; TrialScreen https://app.trialscreen.org/trials/phase-2-safety-efficacy-argx-119-adult-patients-amyotrophic-lateral-sclerosis-trial-nct06441682, accessed on 13 March 2026).

## 3. Clinical and Biomarker Changes in Muscle in ALS

**Figure 4:**
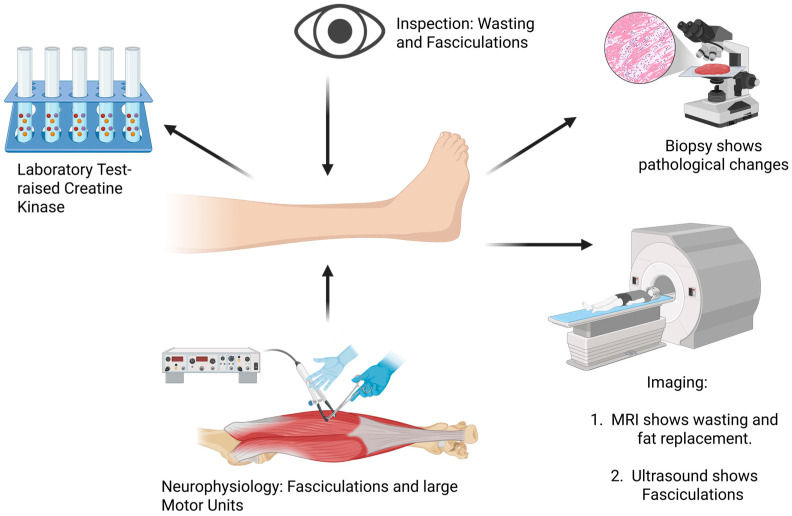
Multi-modal biomarker assessment in ALS muscle pathology. Schematic illustration of biomarker modalities for assessing muscle pathology in amyotrophic lateral sclerosis (ALS). The central limb represents clinical muscle weakness and wasting in patients with ALS. (**Top Left**) Biochemical biomarkers—elevated creatine kinase (CK; ~50% of patients), increased serum lactate, and altered myokines (irisin and FGF21). (**Top Center**) Clinical examination—visible atrophy, weakness, and fasciculations. (**Top Right**) Histopathology—muscle biopsy revealing fiber type grouping, denervation atrophy, inflammatory infiltrates (CD68+ macrophages and CD4+ T cells), COX-negative fibers, and abnormal protein accumulation (TDP-43 and SOD1). (**Bottom Right**) MRI—progressive muscle atrophy, fatty infiltration, and increased T2 signal indicating acute denervation; quantitative MRI tracks disease progression. (**Bottom Left**) Electrophysiology—EMG showing fasciculations, fibrillations, and enlarged motor units; neuromuscular junction instability with decremental responses; and motor unit number estimation (MUNE) documenting progressive motor unit decline. These complementary modalities enable comprehensive assessment of ALS muscle pathology for diagnosis, disease monitoring, and therapeutic evaluation. Created in BioRender. Yarlagadda, S. (2026) https://BioRender.com/66ho01r (accessed on 9 March 2026).

In ALS, clinicians can find changes in muscle with clinical examination, biochemical testing, neurophysiology, MRI (Figure 4) and by histological examination. These examinations reveal that patients with ALS have muscle wasting (atrophy) and weakness [1], with little to no evidence of myonecrosis or fibrosis [18]. By the time of diagnosis, there has been a significant loss of α-MNs, with a corresponding loss of motor units, with compensatory collateral sprouting of motor axons from surviving α-MNs (i.e., axons from intact motor units) occurring to mask clinical muscle weakness [92]. This can be seen as muscle fasciculations, which are thought to occur from axonal or motor neuron hyperexcitability due to muscle denervation [93] and are also typical of ALS [93]. Fasciculations can also be studied with ultrasound [94]. The cell and molecular bases for these changes in ALS muscle most likely stem from a loss of neuromuscular connections, driven in part by invasion of terminal Schwann cells into the synaptic cleft of the NMJ, along with the dispersal of key proteins from the NMJ’s postsynaptic membrane, such as acetylcholine receptors (AChRs) and MuSK [18,95,96]. The latter is a key tyrosine kinase receptor responsible for stabilizing NMJs (Figure 3; ref. [76]). These visible changes in muscle are fundamental clinical features of ALS and are essential for diagnosis.

A mild to moderate rise in creatine kinase (CK), a muscle enzyme important in energy production, is seen in roughly half of patients with ALS [97]. The mechanism for the rise in CK is not entirely clear but is thought to reflect leakage across a damaged sarcolemma following motor neuron degeneration [98]. Some studies have suggested that an elevated CK level is associated with a better prognosis, suggesting that upregulated muscle metabolism in response to physiologic stress could also be responsible for the rise in this enzyme [99,100]. If true, a rise in muscle CK could also be linked to hypermetabolism seen in patients with ALS [101] and perhaps linked to the ALS-risk gene glutathione peroxidae 3 (GPX-3, see Section 1) [11]. However, measurement of CK is frequently part of the investigations of patients with ALS. It is important to know that raised CK can occur in ALS, and that raised CK does not always indicate a primary muscle disease.

Neurophysiology is widely used to study ALS. With needle electromyography (EMG), the findings are those of denervation and re-innervation and not those of muscle damage [102,103]. Typical changes include fasciculations, fibrillations and large motor units. In ALS, there is also evidence of instability of the NMJ, with a decrement seen with repetitive stimulation [90,104]. Supporting these neurophysiological changes are biopsy studies of ALS muscles that revealed decreased quantal content (i.e., decreased levels of evoked neurotransmitter release) in ALS NMJs [105], along with evidence of morphological and ultrastructural studies of ALS NMJs [18,96] (discussed further below). Neurophysiological techniques of motor unit number estimation (MUNE) have been used to show that motor unit numbers decline over time [106,107]. One MUNE study found that motor unit numbers decline in an exponential fashion [108]. MUNE techniques are expected to give information about the rate of loss of motor units and provide a means of tracking disease.

MRI techniques have been applied to the study of muscle in ALS. MRI of muscle in patients with ALS shows atrophy and infiltration with fatty tissue [109,110,111]. With quantitative MRI studies, the proportion of fat in ALS muscle increases over time and is a marker of disease progression [112]. Progressive loss of muscle volume over time is also able to be measured with MRI [112,113]. In the muscle in the early stages of ALS, there can be an increased T2 signal that is thought to be due to edema of acute denervation [114]. MRI has also been used to study fasciculations [115], and MR spectroscopy has been used to investigate biochemical changes in muscle [116]. These techniques have much to offer the study of muscle in ALS, and with advanced MR spectroscopy, it is expected that the metabolism of muscle can be studied.

## 4. Histological Changes in Muscle in ALS

Our knowledge of the pathological features of ALS muscle comes from muscle biopsy studies, including our own recent research [18]. Currently, biopsies are studied for morphological changes, changes in muscle fiber type, fiber type grouping, inflammation and expression of abnormal proteins. The studies reported here have been performed on patients with a diagnosis of ALS. We are not aware of studies on muscles that are specific to the different ALS genes. We propose that modern studies of the pathology of muscle are essential to the understanding of ALS of all types.

### 4.1. Morphological Changes

The first muscle biopsy was reported in 1856, in a report that has been translated from German [117]. After that, there were subsequent publications in the German literature seeking to document muscle morphology in various diseases. In 1953, knowledge was consolidated in an important textbook; this contained a chapter on experimental pathology, outlining the changes that occur after denervation and re-innervation of a muscle [118].

Later, there were reports of a series of muscle biopsies from subjects with ALS. A large series in 1967 described the pathological appearances in biopsies from 348 patients with a range of neuromuscular diseases, including 24 subjects with ALS [119]. It was anticipated that ALS muscle would show changes associated with denervation and re-innervation. Overall, reports of the pathological findings in ALS biopsies indicate that the typical features include changes in denervation, such as small angulated fibers, increased internal nuclei, and pyknotic nuclear clumps, and re-innervation, such as compensatory muscle fiber hypertrophy, target fibers and fiber type grouping [18,120,121,122]. These muscle changes are not specific to ALS and are seen in other neurologic conditions that result in neurogenic atrophy [120].

In Table 1, we have reviewed the previously published series of muscle biopsies in ALS. Biopsies were compared to control (normal) muscle or to other diseases such as spinal muscular atrophy, Charcot–Marie–Tooth disease and other disorders. All the studies reported grouped atrophy/denervation. Some reported predominance of Type I fibers/loss of Type II fibers [18,105,120,122], although others found no change in the ratio of Type I to Type II fibers [123]; others described atrophy of Type I fibers [124,125]. It is possible that Type I fiber atrophy would be prominent if Type II fibers had already degenerated. Table 2 summarizes the techniques that were used and the methods of quantitation. The total number of patients with ALS in these reports is 499, which is a low number considering the diversity and heterogeneity of patients with ALS. The techniques used for the study of muscle biopsies have been conventional histological techniques, and there is scope for more advanced studies.

### 4.2. Changes in Fiber Type

There have been many studies of muscle fiber types in ALS. These are critical to understanding the selective vulnerability of different types of motor units. These studies include observations in rodent models and in humans. There are some limitations in the use of rodent models, but overall, the gross anatomical arrangement of limb and non-limb muscles is similar. However, regarding fiber type, mouse muscle has more type IIb fast glycolytic fibers and faster twitch than human muscle. They also have differences in myosin heavy chains and membrane excitability [36,133,134]. These differences are attributed to the small size of mice compared to humans and the different basal metabolic rate of humans compared to mice [135]. These differences must be kept in mind when considering these studies.

It is thought that the muscle fibers and their motor neurons in ALS show a differential vulnerability to denervation. Much of this evidence comes from multiple rodent studies of ALS. For example, SOD1 and inducible TDP-43 ALS mouse models have demonstrated an early and selective loss of fast-twitch muscle fibers even before motor symptoms are manifest [136,137,138,139,140,141]. In the later stages of disease, several SOD1 mouse models have demonstrated a transition from fast to slower muscle fibers in *tibialis anterior* [139,142,143,144], medial *gastrocnemius* [139,143], *plantaris* [142] and *extensor digitorum longus* [145] muscles, but not in *soleus* [143], a slow-twitch muscle. However, one study of SOD1^G93A^ mice with late-stage ALS found the opposite phenomenon in *soleus* muscle: a shift from slow to fast muscle fiber types [146].

There is also some evidence for a similar pattern of muscle fiber loss in human ALS. This has been shown with neurophysiology and with pathological studies. Selective vulnerability of fast-twitch muscle fibers in 20 patients with ALS was suggested in one electrophysiologic study that demonstrated a preferential loss of larger motor units [147]. A preferential loss of Type II muscle fibers compared to healthy controls was found in *palmaris longus*, *flexor carpi radialis* and *vastus medialis* in an autopsy study of nine patients with ALS [120]. Our own study showed increased Type I fibers in the *vastus lateralis* of ALS subjects [18].

In the only longitudinal human study, there was a trend towards the loss of Type II muscle fibers and an increase in the amount of Type I muscle fibers in the *vastus lateralis* muscle belonging to five patients with ALS over a 12-week period, although this was not statistically significant [122]. One small needle biopsy study of the ALS *vastus lateralis* muscle found that the presence of hybrid fibers was associated with slower disease progression [148]. However, in two large older case series, a predominant loss of Type II muscle fibers was not seen on cross-sectional analysis of *vastus lateralis* in patients with ALS compared to controls [123,131]. This finding is interesting given that fast-twitch fibers have been found to constitute about 70% of the *vastus lateralis* muscle [149]. The early loss of fast-twitch fibers in ALS could explain why the *tibialis anterior* muscle, which is almost exclusively composed of fast-twitch fibers [150], is disproportionately affected in patients with ALS involving the legs [151]. However, human studies looking at the alteration of muscle fiber types within ALS-affected *tibialis anterior* muscle are lacking. Overall, these human studies suggest that in humans, there is an early preferential loss of fast muscle fibers. Understanding how this comes about could provide a further clue to the causes of degeneration in ALS.

Extraocular muscles (EOMs) are relatively spared in ALS. There are several possible explanations for this, including the very rich innervation of these muscles. In addition, unlike skeletal muscles, which usually contain three muscle fiber types, the EOMs contain at least six different muscle fiber types [152]. These include developmental muscle fiber types, embryonic and neonatal, that are known to have slower twitch properties, and moreover, muscle fibers belonging to the EOMs often contain more than one type of myosin heavy chain isoform [152]. An autopsy study of EOM samples from eight patients with ALS showed relatively well-preserved muscle fiber type composition and minimal fiber type grouping compared to age-matched controls, except for a greater loss of slow tonic fibers and near absence of embryonic fibers [153], while other studies have reported altered muscle fiber type distribution within EOMs, such as reduced proportions of myosin heavy chain IIA fibers, in patients with ALS [154,155]. Understanding why the extraocular muscles are spared could also shed light on the processes that lead to degeneration.

### 4.3. Inflammation in Muscle

There is increasing emphasis on inflammation in ALS muscle, which is likely to be important because there are many possible therapeutics that could target inflammation. Several studies have shown inflammatory infiltrates consisting mainly of macrophages within the skeletal muscle of symptomatic SOD1^G93A^ and TDP-43 ALS model mice [156,157,158,159]. Increased expression of macrophage markers, including CCL2, CD68 and CD11b, has also been found in skeletal muscle of symptomatic ALS model mice (SOD1 and TDP-43) [157,158,159]. Macrophage-mediated inflammation appears to increase with disease progression in these model mice, being greatest in end-stage diseases [157,158,159]. However, the presence of macrophages in skeletal muscle is not uniform across muscles, with more macrophages found in the *tibialis anterior* than in the diaphragm [156]. While evidence is limited, it appears that the presence of macrophages contributes to degeneration in these laboratory models.

Regarding NMJs in ALS, macrophages appear to localize around innervating axonal terminals of the NMJ, suggesting they could play a direct role in muscle denervation [156,157,159]. Indeed, recent studies by Nógrádi and co-researchers have shown that CCL2-positive macrophages become localized around NMJs in patients with ALS and ALS model mice (TDP-43, TDP-43^A315T^, TDP-43^M337V^), and that injections of CCL2-neutralizing antibodies can ameliorate NMJ denervation in mutant TDP 43 ALS model mice [159]. These researchers go on to suggest that the CCL2-CCR2 signaling is an inflammatory axis working in muscle to drive the loss of neuromuscular connections in patients with ALS [159]. Interestingly, one study found a correlation between macrophage inflammatory activity and disassociation of terminal Schwann cells from the motor nerve terminal at the NMJs in SOD1^G93A^ rats [157]. This disassociation might in turn allow for direct interaction between CCL2-positive macrophages and the motor nerve terminal for terminal removal by phagocytosis: an idea that remains to be tested. By contrast, it is also possible that the macrophages have a protective role by removing cellular debris and assisting nerve regeneration [156]. Indeed, this beneficial effect of macrophages has been shown in peripheral nerve injury using transgenic mice [160]. Further work is required to understand these opposing possibilities.

Inflammatory infiltrates of both macrophages and lymphocytes have also been seen in the skeletal muscle of some patients with sporadic ALS at varying stages of disease [98,121,122,159]. Where immunohistochemistry has been performed, the lymphocytes were mainly T lymphocytes of the CD4 type [121,161]. Furthermore, increased expression of leucocyte (CD4) and macrophage (CCL2 and CD68) markers has been found in the skeletal muscle of patients with sporadic ALS when compared to controls [122,159].

Increased activation of the innate immune system within skeletal muscle could also be important in the pathogenesis of ALS. Inhibition of the terminal complement pathway using SOD1^G93A^ ALS mice that lack C5a receptor 1 (C5aR1), a key complement protein involved in chemoattraction, appears to reduce macrophage infiltration into skeletal muscle, reduce denervation and improve hindlimb grip strength [158]. Similar findings were also observed in SOD1^G93A^ ALS mice and rats when treated with a C5aR antagonist [162,163,164]. C5a -C5aR1 signaling is also a potent activator of the NLRP3 inflammasome, a pathway that leads to increased expression of pro-inflammatory cytokines such as IL-1β [135]. Indeed, researchers have found increased expression of IL-1β, along with its effector molecules caspase 1 and Asc, within the skeletal muscle of symptomatic SOD1^G93A^ ALS mice and patients with sporadic ALS [165]. There are many therapeutic agents that target complement, so it is important to fully characterize the role of complement in ALS muscle. Collectively, these findings suggest that muscle inflammation is a key early event in ALS pathogenesis, an attractive notion that remains to be substantiated [158,159,165].

### 4.4. Expression of Abnormal Proteins in ALS Muscle

The pathological features of ALS include aggregation of insoluble protein within neurons [166]. Most of the genes that cause ALS encode for proteins or polypeptides that accumulate within cells or are involved in the metabolism of protein aggregates [2,167]. In the majority of patients, there is accumulation in neurons of Tar DNA-binding Protein 43 (TDP-43) plus other proteins, but a small group has accumulations of superoxide dismutase 1 (SOD1) [2]. This aggregation is thought to be a critical part of the pathogenic process.

Although aggregation of insoluble proteins is best shown in neurons, there have also been studies in muscle [6]. There have been some studies showing the accumulation of TDP-43 in the nuclei of ALS muscle [168], as well as in its cytoplasm [169,170]. TDP-43 accumulation in the intramuscular nerves is also described as a marker of ALS [171]. Dipeptide repeats have been found in muscle biopsies of 18 of 37 patients with ALS associated with C9orf72 repeat expansions [172]. SOD1-associated disease differs from other forms of ALS and shows SOD1 accumulations in neurons. SOD1 has also been shown to aggregate in skeletal muscle cells in the hindlimbs of mutant SOD1^G93A^ and SOD1^G37R^ ALS mice, which in turn can lead to severe muscle pathology, including loss of NMJs [173,174]. However, in the muscles of patients with ALS carrying SOD1 mutations, such an accumulation has not been found [175,176]. Accumulation of abnormal proteins in muscle would suggest that muscle is a primary target of the pathological processes of ALS, but more studies are required.

### 4.5. Mitochondria in ALS Muscle

Functional abnormalities of mitochondria are present in muscle in ALS [121,177], as evident by increased numbers of cytochrome c oxidase (COX)-negative muscle fibers (COX, also termed Complex IV, is a key enzyme in the mitochondrial electron transport chain) [121,178]. However, one study of 36 patients with sporadic ALS did not find the ratio of COX-negative muscle fibers to be significantly greater than that seen in muscle from 69 controls [179]. Ragged red fibers, which also signify mitochondrial dysfunction, have been described in some cases of ALS [121,180]. Ultrastructural studies have also found abnormalities of mitochondria in ALS muscle, with accumulation of abnormal mitochondria in the subsarcolemmal region of muscle and proximal axons of anterior horn motor neurons in patients with sporadic ALS [181,182,183]. These pathological findings are consistent with the observations of biochemical changes in mitochondrial dysfunction in biopsied muscle [184] and of higher serum lactate concentrations at rest and during exercise in those with sporadic ALS compared to healthy controls [185,186]. Other studies have shown reduced expression of networks involved in mitochondrial activation [187]. At the genetic level, damage to mitochondrial DNA, including deletions, has been observed more frequently in the muscle of patients with sporadic ALS, although the amount of deletions is considerably less than that seen in those with mitochondrial disease [110,179,188]. Mitochondrial damage is therefore likely part of the degenerative process in muscle in ALS.

### 4.6. Replacement of Muscle with Fat

After denervation, there can be replacement of muscle fibers with fatty tissue, as can be seen with chronic neuropathies [189]. This replacement also appears to be a consequence of the denervation of ALS. Routine clinical MRI and high-field MRI show increased fat in ALS muscle [190]. Little is known about the cellular process of fat accumulation in ALS muscles. However, in aging, when myosteatosis is common, the process involves the replacement of myofibers with adipocytes [191]. Before replacement with adipocytes, lipid can be stored as lipid droplets, both within and between muscle fibers [192], and this process has been observed in ALS muscle [193]. Such pathology is also seen in some animal models of ALS, such as the TDP-43^Q331K^ transgenic mouse [194]. Fat accumulation can therefore be a marker of ALS. It could occur because of metabolic changes or could indicate that, in ALS, there are degenerative changes similar to those of aging.

### 4.7. Neuromuscular Junction in ALS

NMJs are infrequently observed in routine muscle biopsies. However, when biopsies are performed at the site of motor endplates, denervation and disassembly of the NMJ can be observed [18,96,195]. The alterations to the motor endplate are accompanied by invasion of terminal Schwann cell processes into the synaptic cleft of the NMJ [18,96] and complement deposition [196]. Denervation is accompanied by re-innervation and the formation of new NMJs, termed collateral re-innervation. It occurs by the sprouting of terminal motor axons [84,92], resulting in motor unit expansion and fiber type grouping [18,96]. During this process of re-innervation, terminal Schwann cells play a role in guiding the motor axonal sprouts to the site of the denervated NMJ [197]. Table 3 summarizes the reports of the pathology of NMJs in ALS. For the papers that gave numbers of patients, these are from 59 patients, which is a small number, considering the heterogeneity of ALS. However, these studies consistently demonstrate NMJ pathology in ALS.

While it is unclear whether changes at the NMJ are driven by denervation or by changes in muscle, it would be expected that pathological changes at the NMJ could occur before overt weakness, since there can be loss of muscle fibers before weakness occurs [92]. However, in mouse models of ALS (SOD1^G37R^ [84] and TDP-43^Q331K^ [71]), there is evidence that pathological changes at the NMJ can occur before loss of motor units. This would indicate that pathology at the NMJ could occur first and lead to withdrawal of innervation and the loss of motor units. Experiments show that expression of mutant SOD1^G93A^ in mouse muscle leads to disintegration of the NMJ through mechanisms involving protein kinase C [136,173,174]. In NMJs formed in vitro from muscle and nerve cells derived from human stem cells, there were abnormalities in NMJs from ALS compared to controls [18,202]. These abnormalities would be evidence for the dying back theory of ALS and could support an approach to protect the NMJ as a therapeutic strategy.

In the mutant SOD1^G93A^ mouse model, studies have shown the importance of MuSK (Muscle-specific tyrosine kinase) in the degeneration of nerve fibers. Either overexpression [86] or antibody stimulation [89] of MuSK, which is necessary for the growth and stabilization of AChRs at the NMJ (Figure 3) (see above), can lead to preservation of NMJs. However, although treatment with an antibody agonist to stimulate MuSK can lead to preservation of NMJs, some studies have shown that this stimulation did not improve the strength of the diaphragm—a key muscle affected in ALS [203]. In other ALS models, such as a mouse model of C9orf72-related ALS, a MuSK agonist led to preservation of the NMJ [90]. Collectively, these studies suggest some promise for peripheral therapeutic treatment of muscle in ALS in stabilizing the NMJ. However, these are studies in animal models that may not be applicable to the human disease, where most patients do not carry ALS-associated genes.

In ALS, there have been some investigations of the other NMJ-associated proteins such as LRP4, the receptor for neural-agrin—a protein released by the motor nerve—and DOK7 (a MuSK effector protein; See Figure 3). Neural-agrin binds to LRP4, which activates MuSK, which in turn activates DOK7. Both MuSK and DOK7 are needed to trigger the formation and maintenance of the NMJ [204,205] (Figure 3). Our study of human muscle found less LRP4 and DOK7 in patients with ALS compared to controls [18], and antibodies to LRP4 have been found in the serum of patients with ALS [206,207]. The functional consequences of these changes in humans have not yet been studied, but one study found that adenovirus-directed increase in the expression of DOK7 led to preservation of the NMJ in mutant SOD1^G93A^ mice [87]. The effects of increasing LRP4 in mutant SOD1 ALS model mice are not yet known.

Recent research employing stem cell-derived motor neurons carrying ALS-linked FUS mutations has also revealed a downregulation of proteins needed for NMJ neurotransmitter release and adhesion (e.g., SNAP 25, Ephrin receptor 4aA, and laminins-α1 and -β1 [208]), but again, the functional consequences are unknown, and the relevance of this finding to human disease is unclear. Further research on the role of the NMJ in ALS will help to understand whether NMJ breakdown can originate with pathology in muscle cells and whether this is a target for ALS therapy.

### 4.8. Satellite Cells in ALS

There are a few morphological studies of satellite cells in human ALS muscle. One small human study reported “lack of activation” of satellite cells in muscle biopsy [122]. However, it is likely that satellite cells play a role in ALS [79]. Satellite cells are necessary for muscle regeneration, and some studies have suggested impaired satellite cell function in ALS [209,210]. Indeed, there is mounting evidence that muscle satellite cells contribute to homeostasis of the NMJ and that alterations in motor neuron health, including their activity, can have adverse effects on this cell population and their proposed neuromuscular functions [211,212,213,214,215]. In support of this idea, ALS researchers have shown that there is variation in the transcriptional profiles and renewability of skeletal muscle satellite cells from vulnerable versus resistant muscles in ALS model mice. For example, in mutant SOD1^G93A^ ALS model mice, extraocular muscle satellite cells contain more transcripts for axonal guidance molecules and thus have greater renewability than muscle satellite cells from diaphragm and hindlimb muscles [216]. This observation may in part account for diaphragm and hindlimb muscles being more prone to ALS-like diseases compared to extraocular muscles. The applicability of these findings in animal models to human ALS remains to be clarified. However, the possibility of enhancing satellite cell function to protect ALS muscle is attractive and worth further study.

## 5. Metabolic and Molecular Changes

### 5.1. Metabolic Reprogramming

Metabolic reprogramming, when there is a shift in energy metabolism in response to changes in the environment, occurs in many tissues under physiological stress. It has been well studied in cancer biology [217] and immunology [218] and has been described in neurological diseases [219]. There have been studies of this process in transgenic animal models of ALS. Metabolic reprogramming has been described in the spinal neurons from 12.5-day-old mutant SOD1^G93A^ ALS model mice [220] and in the spinal cord in mutant SOD1^G93A^ mice, with a shift to the use of fatty acids as an energy source [144,221]. In studies of muscle in ALS, studies in mutant SOD1^G93A^ and SOD1^G86R^ rodent models have shown increased oxidative metabolism in the *tibialis anterior*, before the onset of motor symptoms [144,222]. The early decrease in glycolytic metabolism is paralleled by an increased reliance on lipid metabolism [144,222]. Rescue of glycolytic metabolism by pharmacologic inhibition of fatty acid β-oxidation in the *tibialis anterior* muscle of mutant SOD1^G93A^ and SOD1^G86R^ mice has been shown to result in preserved muscle strength [144] and weight gain [222] compared to controls, but effects on survival were dependent on complete or partial inhibition of fatty acid β-oxidation [144]. Similarly, improved metabolic function after administration of trimetazidine leads to increased survival of mutant SOD1^G93A^ mice [223]. A study of symptomatic rodents that only expressed SOD1^G93A^ in muscle also demonstrated a shift in the metabolism of fast-twitch *tibialis anterior* muscle from glycolytic to oxidative, suggesting that metabolic reprogramming of muscle in ALS might occur independent of motor neuron degeneration [224]. However, the applicability of studies in these models is uncertain, particularly since SOD1 variants are found in only a minority of ALS subjects.

In humans with ALS, the metabolic perturbation of skeletal muscle is less clear. One study found that Pyruvate Dehydrogenase Kinase 4 (PDK4), a mitochondrial enzyme involved in switching cells from glycolytic to lipid metabolism, is upregulated in the skeletal muscles of patients with sporadic ALS, as well as SOD1^G86R^ ALS model mice [222]. In human primary myotubes grown in culture, there is evidence of increased dependence on the use of fatty acids as a fuel substrate [101]. However, a proteomic analysis of *tibialis anterior* muscle taken from patients with sporadic ALS found evidence supporting a reduction in both glucose and lipid metabolisms [225]. A study of muscle progenitor cells found that FOXO1 is a factor that drives muscle damage and metabolic switch [226]. Recent studies have shown that, as well as changes in mitochondria, the switch to fatty acid metabolism is associated with alterations in cholesterol transport [227]. Thus, there is some evidence of metabolic shift in human ALS muscle, but the studies are limited, and more work is required, since there are therapeutic agents that impact metabolism.

### 5.2. Myokines, Neurotrophins and Muscle Growth Factors

Muscle produces many factors that act both locally and systemically. Alterations in these functions of muscle could play a role in ALS, but so far, this has not been studied extensively. In response to cellular stress, muscle cells increase the production of myogenic regulatory factors (MRFs) such as MyoD1, MyoG and Myf5 [228]. MRF mRNA was found to be upregulated in the skeletal muscle of mutant SOD1^G93A^ rodents during the later stages of disease, suggesting their role in myogenesis [229]. This work is limited, being on only a single disease model. There are yet no studies of these factors in human ALS muscle. However, these important factors play a role in muscle response to injury and deserve further study.

Muscle is known to secrete molecules (myokines) in response to muscle activity [230,231,232]. There have been some studies of myokines in ALS. Irisin is a myokine that plays a role in energy metabolism and immunity [233]. Levels of Irisin are increased in patients with hypermetabolism in ALS and correlate with disability [234]. Fibroblast growth factor 21 (FGF21) is a myokine that shows alteration in ALS muscle, with increases seen in atrophic fibers in humans and SOD1^G93A^ mice, and elevated plasma levels being associated with slower progression of ALS [235]. Fibroblast growth factor binding protein 1 (FGFBP1) is secreted by muscle fibers and assists in the maintenance of NMJs. Secretion of FGFBP1 is reduced in the muscles of SOD1^G93A^ mice [236]. Insulin-like growth factor 2 (IGF-2) is another muscle growth factor which appears to play a role in muscle survival in ALS. For example, in SOD1^G93A^ ALS mice, the levels of IFG-2 and its receptor IGF-1R are maintained in oculomotor nerves and their target muscle, extraocular muscles—muscles which are relatively resistant to ALS pathology [237]. These studies need to be replicated, and the significance of these changes needs further exploration, but changes in myokines could be a mechanism by which changes in muscle lead to metabolic changes in ALS.

Denervated muscle fibers express neurotrophic factors that play a role in re-innervation by terminal branches of motor axons [238]. One such factor is Neurturin that is produced by muscles and promotes motor neuron recruitment and formation of NMJs [239]. Treatment with Neurturin has been beneficial in providing neuroprotection in animal models of ALS (SOD1^G93A^ mice) [240]. It is not clear whether ALS muscle produces Neurturin, but this factor has obvious interest as a molecule involved in the formation of NMJs and could be a possible therapeutic agent.

### 5.3. Gene/microRNA Expression

**Figure 5:**
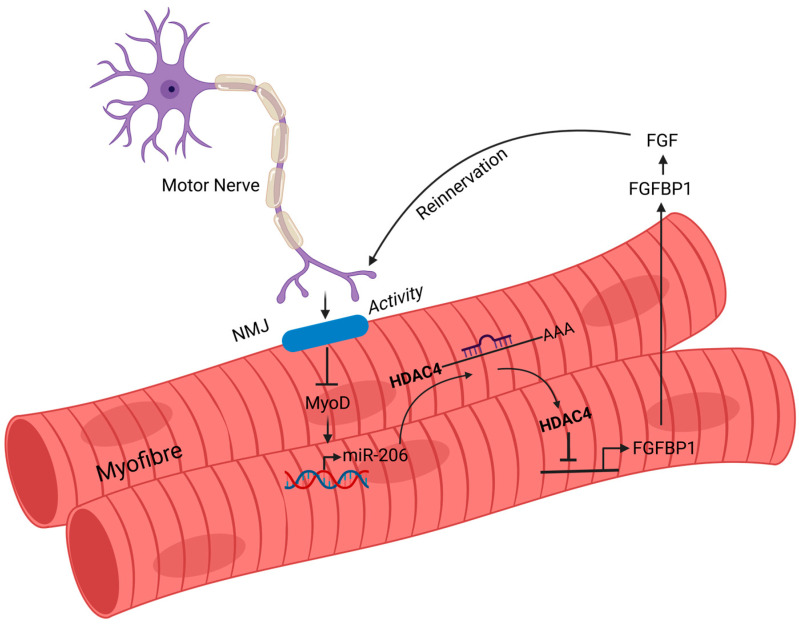
miR-206 signaling in ALS skeletal muscle. In ALS, skeletal muscle shows both degenerative changes and compensatory repair mechanisms. One key molecular response involves the upregulation of miR-206, a muscle-specific microRNA that is associated with muscle regeneration. MyoD transcriptionally activates miR-206 following denervation and promotes re-innervation by suppressing histone deacetylase 4 (HDAC4). Reduced HDAC4 activity leads to increased expression of fibroblast growth factor binding protein 1 (FGFBP1), which enhances FGF signaling and supports synaptic repair. This pathway has been shown to delay disease progression in SOD1^G93A^ mouse models, highlighting miR-206 as a potential therapeutic target. Adapted from Williams et al., 2009 [244], and created in BioRender. Yarlagadda, S. (2026) https://BioRender.com/7n5l6hc (accessed on 9 March 2026).

In ALS muscle, there are changes in gene expression that are due to the disease and attempts at repair [122]. This can be seen at a molecular level with increased expression of microRNAs involved in muscle regeneration [187]. Most of these studies come from animal models, and the relevance to human ALS is uncertain. There is an alteration in the expression of some microRNAs in muscle in ALS, and it has been suggested that this contributes to pathogenesis [241,242,243]. For example, microRNA miR-206 delays ALS progression in mutant SOD1^G93A^ mice [244]. This effect of miR206 is in part through its regulation of histone deacetylase 4 and subsequent fibroblast growth factor signaling pathways [95,244,245] (Figure 5). One study shows that the suppression of microRNA-23a can suppress disease in a mouse TDP43 model (rNLS8 TDP-43 [246]).

One emerging mechanism in the pathogenesis of ALS is altered RNA metabolism, brought about in part by the loss of function of DNA/RNA binding proteins, such as TDP-43, and its ability to regulate alternative splicing and RNA processing of critical genes needed for synaptic function (e.g., STMN2 and UNC13As [247,248,249]). Such loss of function is thought to be due to perturbed transport of TDP-43 into the neuronal nucleus [250,251]. Regarding skeletal muscle, there is accumulating evidence that this function of TDP-43 is critical for regulating the expression of genes needed for myogenesis, including the proliferation and differentiation of muscle progenitor cells ((e.g., skeletal α-actin, Pax 7 and MyoD) [252,253,254]). Indeed, depletion of TDP-43 from muscle nuclei and its accumulation and phosphorylation in the cytoplasm have been observed in the muscles of some patients with ALS [169,254]. In TDP43 mutant mouse models, the cytoplasmic accumulation of TDP-43 is associated with a progressive loss of muscle function and subsequent denervation, which in part can be rescued by suppression of cytoplasmic TDP-43 [255]. Despite these intriguing observations, the role of TDP-43 in controlling specific muscle gene splicing events and the process of their mRNAs in ALS muscle remains an open area of research in need of full pathological characterization in ALS muscle.

### 5.4. Extracellular Vesicles

**Figure 6:**
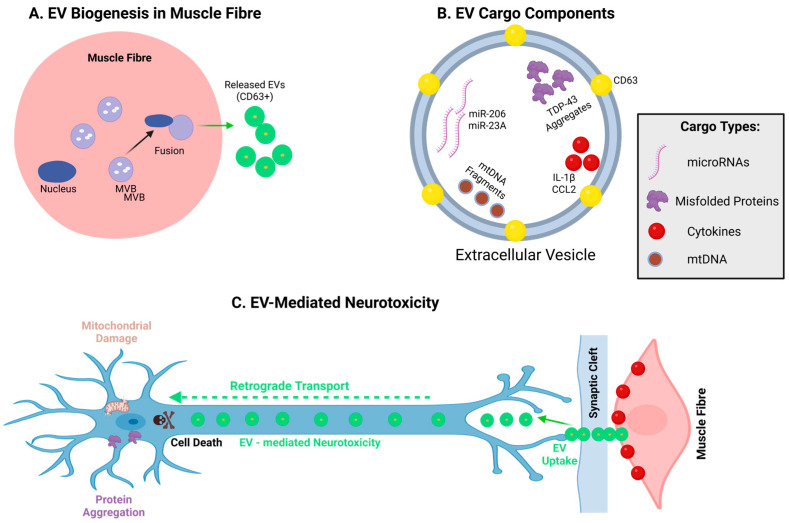
Extracellular vesicle-mediated neurotoxicity in ALS muscle. EV biogenesis, cargo, and pathogenic effects in ALS. (**A**) Multivesicular bodies (MVBs) fuse with the muscle fiber membrane, releasing CD63+ EVs. (**B**) EVs contain pathological cargo: misfolded TDP-43, microRNAs (miR-206, miR-23A), mitochondrial DNA, and inflammatory cytokines (IL-1β, CCL2). (**C**) EVs are taken up at the neuromuscular junction and undergo retrograde transport to motor neurons, where toxic cargo induces protein aggregation, mitochondrial damage, and neuronal degeneration, contributing to ALS pathogenesis through muscle-to-neuron signaling. Created in BioRender. Yarlagadda, S. (2026) https://BioRender.com/7w77vxu (accessed on 9 March 2026).

There has been recent interest in the role of extracellular vesicles in ALS. One electron microscopic study of human ALS muscle found an increase in multivesicular bodies filled with exosome-like structures, which were shown to stain with antibodies to CD63, a marker of vesicles. When extracted, these vesicles were found to be toxic to motor neurons [256]. Extracellular vesicles (EVs) are found to be important in other aspects of ALS, such as the transfer of misfolded proteins and microRNAs [257,258]. Recent research has shown that ALS muscle can secrete EVs that are taken up by the innervating α-MN to trigger death [256,259] (Figure 6), but more work is required to fully assess the role of muscle EVs in ALS. Nevertheless, EVs offer a means by which pathology can spread from cell to cell.

### 5.5. ALS Modeling Using Neuromuscular Organoids and ALS Proteomics

The use of muscle organoids or cells with and without α-MNs has been best developed for muscle diseases with a known single genetic mutation, such as Duchenne muscular dystrophy (mutated dystrophinin; [260,261]) and congenital myasthenia gravis (genetic mutations to genes that encode for AChRs and members of its clustering pathway, such as MuSK, LRP4, DOK7 and Rapsyn; see Figure 3 [262]). These two disorders are diseases of the developing neuromuscular system. By contrast, ALS is a neural degenerative disease of the adult neuromuscular system, which, even in ALS mammalian models, manifests post the fetal to adult switching of molecular components of the neuromotor system. These include the following. For α-MNs synapses, potassium–chloride transporter switches (NKCC1 to KCC2). This developmental switch allows for glycinergic neurotransmission to convert from being depolarizing (excitatory) to inhibitory (hyperpolarizing) [263]. Thus, during development, α-MNs are largely governed by excitation, and then later, when pattern output of neural activity is required, by a combination of excitation (glutamatergic and cholinergic) and inhibition (Glycinergic) [264,265]. There are also fetal to adult neurotransmitter receptor switches of glycinergic receptors that aid in the maturation of glycinergic neurotransmission [266].

At the NMJ, there is pre- and postsynaptic molecular switching that occurs post birth in mammals (including humans). At the pre-synaptic membrane N to P/Q voltage-gated calcium channels, switching allows for enhanced efficacy of synaptic transmission [70,267]. During this period, in the muscle’s postsynaptic region, there is a switch in acetylcholine receptor units, from gamma to epsilon [268]. This switch allows for faster modulation of postsynaptic depolarization that will, in turn, govern the generation and frequency of muscle action potentials. These events largely occur during the loss of polyneuronal innervation of muscle post birth. For muscle, this developmental period is accompanied by the specification of muscle fiber type (fast and slow twitch) and stabilization of the motor unit. Human organoid models, which are generated mostly from adult or embryonic pluripotent stem cells (iPSCs), can be of value to model the effect of a defined mutation in ALS (e.g., see [269] and reviewed by [270]). However, it must be remembered that these models are models of a developing neuromotor system, and not of a mature neural system. The challenge for researchers will be to age these models to display many of the above molecular switches if they are to be translational to ALS (see review by [270]).

In addition, there is the challenge of developing a neuromotor system that carries an epigenetic signature, which is acquired by age and is a signature that is lost when generating models from stem cells [271]. This issue is becoming important in ALS research, where researchers such as Kevin Rhine et al. have recently shown that direct programming of α-MNs from adult fibroblasts retains an aged epigenetic signature when compared to aged human brain neurons, and that this signature is lost in α-MNs generated from iPSCs [271]. Further, as neurons age, they appear to be depleted of RNA-binding proteins, including the ALS-linked protein TDP-43, which becomes accumulated in the cytoplasm [271]. This redistribution of RNA-binding proteins such as TDP-43 is likely to be most important in driving the neural pathology of ALS [272]. Whether such aged TDP-43-dependent defects also occur in ALS muscle is not known. In the future, organoids for ALS research may require direct programming of α-MNs from adult fibroblasts and of muscle cells from adult satellite cells.

At the post-translational level, proteomic studies using control (non-ALS) and ALS muscle biopsies have revealed that arginine methylation is altered in ALS muscle compared to control muscle [273], a disturbance that may explain why patients with ALS become hypermetabolic [101]. These findings provide for future opportunities to explore the function of asymmetric demethylation as a regulator of muscle pathophysiology in ALS.

## 6. Conclusions

In this review, we have described the abnormalities that are found in muscle, focusing, when possible, on human ALS. The role of muscle in ALS is clinically important since the cardinal signs of ALS are muscle weakness and wasting, and death is due to weakness of the respiratory muscles.

The prominent finding in muscle is degeneration that appears to be neurogenic, as seen by the fiber type grouping and the selective early loss of fast fibers. Type II muscle fibers are supplied by FF motor units and are responsible for bursts of movement. The NMJ is degraded early. There is also substantial early regeneration, alterations in muscle metabolism, mitochondrial dysfunction, changes in secretion of soluble factors by muscle, as well as the emerging role of TDP-43 in ALS muscle pathology. These changes have led to suggestions that muscle is a target for therapy in ALS [254]. Indeed, drugs and treatments aimed at improving muscle health and its cellular environment (e.g., muscle satellite and immune cells) have recently been reviewed (see [6,274]).

Further studies of muscle in people with ALS would be welcome. It would be ideal to use modern techniques to confirm the morphological features, to quantify changes in fiber type, to quantify inflammation and examine features that lead to variability in inflammation, and to quantify mitochondrial changes. There is also a need to clarify the role of RNA-binding proteins such as TDP-43 in regulating the expression of muscle genes in ALS muscle, and to investigate the accumulation of lipid in muscle. This would ideally be done at a molecular level with RNA seq and single-cell gene expression.

In genetic studies, it would be useful to study the numerous muscle genes for their effect on susceptibility to disease and as modifiers of outcome. It is well known that pathogenic variants in these genes can lead to weakness and fatigue of muscles, and it can be speculated that variants in such genes could modify the course of ALS. There are no studies of this at present. In terms of therapy, there are opportunities to try to improve muscle metabolism, to improve regeneration and to reduce muscle inflammation. One field that is emerging is therapies directed against complement. There are also avenues to explore the contribution of muscle tissue as a driver of ALS pathology.

## Figures and Tables

**Table 1:** Reports of pathological findings in ALS muscle.

Author, Date	No of ALS Subjects	No of Controls; Type of Controls	Findings in ALS	Refs.
Haase and Shy, 1960	14	17; Charcot–Marie–Tooth disease	Groups of small fibers, 20% showed inflammation.	[126]
Anderson 1967	24	324; various disorders	Small group atrophy, large group atrophy, angulated fibers, abnormal motor endplates.	[119]
Brooke, 1969	182	83; various disorders	Greater atrophy of Type I fibers.	[124,127]
Mastaglia 1971	6	31; spinal muscular atrophy, peripheral neuropathy	Denervation and atrophy.	[128]
Dastur 1973	21	238; muscular dystrophies and other diseases	Denervation changes.	[129]
Achari 1974	111	Nil	Atrophy of single fibers, group atrophy, majority showed denervation, 11 showed mononuclear infiltrate.	[98]
Fidzianska 1976	5	4; early onset spinal muscular atrophy	Atrophy of muscle fibers.	[130]
Telerman-Toppet 1978	18	12; Charcot Marie Tooth disease	Group atrophy, loss of Type II fibers, increased intermediate type fibers.	[120]
Patten 1979	24	Nil	Grouped Type I fibers, Type I fiber atrophy correlated with severity.	[125]
Froes 1987	20	20; healthy controls	Denervation and re-innervation, increased connective tissue, increased variation in fiber size.	[131]
Iwasaki 1991	24	20; healthy controls	No change in the ratio of Type 1 to Type II fibers.	[123]
Maselli, 1993	10	0	Grouped atrophy, Type I fiber predominance.	[105]
Baloh 2007	9	21; various	Grouped atrophy, no change in fiber type proportions	[132]
Al Sarraj 2014	31	20; healthy controls	Atrophy, 6/31 had inflammation, 4/31 had Cox negative fibers, none showed TDP43 pathology.	[121]
Jensen 2016	5	2; healthy controls	Denervation, atrophy increased over 12 weeks, lack of activation of satellite cells, trend to loss of Type II fibers over time.	[122]
Ding 2022	6	3; non-MND	Denervation, increased Type 1 fibers, small fibers in ALS.	[18]

**Table 2:** Method used in Pathological findings in ALS muscle.

Author, Date	Staining Techniques	Methods	Refs.
Haase and Shy 1960	Paraffin sections, H&E, Gomori trichrome	Presence or absence of isolated lesions, group lesions, architectural changes, phagocytosis and inflammation, basophilia and prominent nucleoli, endomysial collagen, increased endomysial fat, increased internal nuclei.	[126]
Anderson 1967	Paraffin & frozen sections, H&E, Gomori trichrome, Bielschowsky, enzyme histochemistry	Scoring of: fiber size, sarcoplasm and nuclei, abnormalities of supporting tissue.	[119]
Brooke, 1969	Frozen sections, ATPase staining	Measurement of fiber size.	[124,127]
Mastaglia 1971	Paraffin & frozen sections. H&E, picro-Mallory and phosphotungstic acid haematoxylm 21 (PTAH). Sudan black (neutral fat), succinic de-hydrogenase, myosin ATPase and acid phosphatase	The number of necrotic fibers, regenerating fibers, fibers with internal nuclei and fibers with enlarged vesicular nuclei in this sample of 100 fibers was then estimated. The severity of other changes such as nuclear clumping and chain formation. Increase in fat and connective tissue and inflammatory infiltrates, degree of grouped atrophy and the numbers of randomly distributed presumably denervated fibers were assessed subjectively.	[128]
Dastur 1973	Paraffin & frozen sections, H&E, Picro-Mallory, enzyme histochemistry, SDH	Measurement of fiber size, assessment of group atrophy, percentage of Type I and Type II fibers.	[129]
Achari 1974	H&E and other stains	Evaluation of changes in fibers, nuclei, and connective tissue; presence of cellular infiltrate.	[98]
Fidzianska 1976	Epoxy sections, methylene blue staining	Assessment of atrophy and degeneration.	[130]
Telerman-Toppet 1978	Methylene blue staining, ATPase and NADH diaphorase staining	Proportions of fibers types, fiber type grouping, atrophy.	[120]
Patten 1979	ATPase and Electron microscopy	Atrophy, group atrophy, fiber type numbers.	[125]
Froes 1987	H&E, Masson’s trichrome, modified Gomori trichrome, NADH-TR, SDH, non-specific esterase, ATPase	Qualitative assessment by recording the presence of central nuclei, split fibers, degenerating or regenerating fibers, structural changes within muscle fibers (targets, ‘moth-eaten’ and ring binden), hypertrophic or atrophic fibers (scattered or grouped), and fiber type grouping. Each parameter was graded from 0 (not present) to ++++ (greater than 50 fibers affected).	[131]
Iwasaki 1991	H&E, Gomori trichrome, ATPase	Qualitative assessment of central nuclei, split fibers, degeneration or regenerating fibers, structural changes within muscle fibers (target, moth-eaten and ring fiber), hypertrophic and atrophic fibers (scattered or grouped). Each parameter was graded from 0 (not present) to +++ (greater than 30 fibers affected).	[123]
Baloh 2007	ATPase staining	The frequency of groups of atrophic fibers was defined by the mean number per high-power field (HPF, 20× magnification). The number of atrophic fibers in each group were counted, and groups were scored as containing either a single fiber type (I or II) or mixed fiber types on myosin ATPase staining.	[132]
Al Sarraj 2014	H&E, ATPase, NADH-TR, SDH, COX, Gomori trichrome, acid phosphatase Periodic acid-Schiff, myo-phosphorylase, Sudan Black. Immune staining for P62, lymphocytes, complement	Examined for small angular fibers, grouped atrophy, fiber type grouping. Scored for presence or absence of neurogenic changes, inflammation, necrosis, COX negative fibers, HLA, C5b-9, P62 and TDP 43.	[121]
Jensen 2016	H&E, myosin heavy chain, PAX7, C68 staining	Quantitation of stained cells.	[122]
Ding 2022	H&E and myosin heavy chain staining	Quantification of fast and slow fibers, fiber type grouping, muscle fiber diameter, small angular fibers.	[18]

**Table 3:** Reports of pathological changes at the NMJ in human subjects with ALS.

Author, Date	No of ALS Subjects	No of Controls; Type of Controls	Type of Sample	Findings in ALS	Ref.
Bjornskov 1975	21 (267 endplates)	5 (120 endplates)	Intercostal muscle biopsy	Enlarged, segmented motor endplates in ALS.	[198]
Bjorn Skov 1984	467 endplates	600 endplates	Intercostal muscle biopsy	Segmented endplates.	[199]
Tsujihata 1984	11 (74 endplates)	0	Biceps brachii muscle biopsy	Loss of nerve terminals in 33% of motor endplates in ALS.	[200]
Yoshihara 1998	4 (Endplate number not specified)	3 normal controls (Endplate number not specified)	Laryngeal muscle from laryngectomy	In ALS: some NMJs showed absent nerve terminal, flattened synaptic clefts and intrusion of Schwann cells.	[201]
Bruneteau 2015	9 (430 NMJs)	0	Deltoid or anconeus muscle biopsy	In ALS, denervation of 19% of endplates, 56% of innervated endplates showed re-innervation, intrusion of terminal Schwann cells into synaptic clefts.	[96]
Bahia 2016	11 (No of endplates not stated)	6 normal controls (No of endplates not stated)	Post-mortem intercostal muscle	Fewer and smaller endplates in ALS, with complement deposition.	[196]
Ding 2022	3 (77 NMJs)	5 non-MNDs (55 NMJs)	*Vastus lateralis* and deltoid muscle biopsies	Smaller motor nerve boutons, loss of nerve terminal area over the motor endplate, which led to a drop in the % of motor nerve terminal to AChR overlap. Evidence of terminal Schwan cell intrusion into the synaptic cleft. De-localization of MuSK from the motor endplate.	[18]

## Data Availability

No new data were created or analyzed in this study. Data sharing is not applicable to this article.

## Abbreviations

The following abbreviations are used in this manuscript:

AChRs	acetyl choline receptors
AMPK	AMP-activated protein kinase
ATP	adenosine triphosphate
α-MNs	alpha-motor neurons
ALS	amyotrophic lateral sclerosis
C5aR1	C5a receptor 1
CK	creatine kinase
EMG	electromyography
EOMs	extraocular muscles
EVs	extracellular vesicles
FF	fast fatigable
FR	fast fatigable resistant
FGFBP1	fibroblast growth factor binding protein 1
FGF21	fibroblast growth factor 21
IGF-2	insulin-like growth factor 2
MUNE	motor unit number estimation
MuSK	muscle specific tyrosine kinase
MRFs	myogenic regulatory factors
MHC	myosin heavy chain
NFAT	nuclear factor of activated T cells
NMJ	neuromuscular junction
PGC1α	peroxisome proliferator-activated receptor-gamma coactivator-1 alpha
PDK4	pyruvate dehydrogenase kinase 4
Ref.	references
SR	sarcoplasmic reticulum
SIRT1	sirtuin 1
SOD1	superoxide dismutase 1
TDP-43	tar DNA binding protein 43
